# Exome scale map of genetic alterations promoting metastasis in colorectal cancer

**DOI:** 10.1186/s12863-018-0673-0

**Published:** 2018-09-19

**Authors:** Krzysztof Goryca, Maria Kulecka, Agnieszka Paziewska, Michalina Dabrowska, Marta Grzelak, Magdalena Skrzypczak, Krzysztof Ginalski, Andrzej Mroz, Andrzej Rutkowski, Katarzyna Paczkowska, Michal Mikula, Jerzy Ostrowski

**Affiliations:** 1 Depatment of Genetics, Maria Sklodowska-Curie Memorial Cancer Center and Institute of Oncology, Roentgena 5, 02-781 Warsaw, Poland; 2 Department of Gastroenterology, Hepatology and Clinical Oncology, Medical Center for Postgraduate Education, Warsaw, Poland; 3 Laboratory of Bioinformatics and Systems Biology, Center of New Technologies, University of Warsaw, Warsaw, Poland; 4 Department of Patomorphology, Medical Center for Postgraduate Education, Warsaw, Poland; 5 Department of Oncological Gastroenterology, Maria Sklodowska-Curie Memorial Cancer Center and Institute of Oncology, Warsaw, Poland

**Keywords:** Colorectal cancer, Metastasis, Exome, Gene expression, Sequencing

## Abstract

**Background:**

Approximately 90% of colorectal cancer (CRC) deaths are caused by tumors ability to migrate into the adjacent tissues and metastase into distant organs. More than 40 genes have been causally linked to the development of CRC but no mutations have been associated with metastasis yet. To identify molecular basis of CRC metastasis we performed whole-exome and genome-scale transcriptome sequencing of 7 liver metastases along with their matched primary tumours and normal tissue. Multiple, spatially separated fragments of primary tumours were analyzed in each case. Uniformly malignant tissue specimen were selected with macrodissection, for three samples followed with laser microdissection.

**Results:**

> 100 sequencing coverage allowed for detection of genetic alterations in subpopulation of tumour cells. Mutations in KRAS, APC, POLE, and PTPRT, previously associated with CRC development, were detected in most patients. Several new associations were identified, including PLXND1, CELSR3, BAHD1 and PNPLA6.

**Conclusions:**

We confirm the essential role of inflammation in CRC progression but question the mechanism of matrix metalloproteinases activation described in other work. Comprehensive sequencing data made it possible to associate genome-scale mutation distribution with gene expression patterns. To our knowledge, this is the first work to report such link in CRC metastasis context.

**Electronic supplementary material:**

The online version of this article (10.1186/s12863-018-0673-0) contains supplementary material, which is available to authorized users.

## Background

High mortality rate of colorectal cancer stems from its metastatic potential [1]. Metastasis is also crucial health problem for other tumours - it causes 90% of deaths for all solid tumours [2]. Recently great progress has been made in the understanding of biological principles of the metastatic process [3], which translated into new therapies extending patient survival over twofold [4]. Further advances in clinical treatment are hampered by genetic heterogeneity and evolutional potential of lesions. Genotyping of single variant or even single whole gene is often insufficient to predict effectiveness of molecularly targeted therapies and we still lack the thorough atlas of underlying genetic aberrations.

The development of primary colorectal tumour (PT) occurs along well described sequence of genomic mutations. The most essential are alterations in APC, TP53, KRAS, PIK3CA and TGFB, but many others have been detected - 46 genes have been causally linked to the development of CRC according to the Catalogue Of Somatic Mutations In Cancer (COSMIC) database [5]. In contrast, no mutations have been associated with metastasis yet [6].

There are two possible reasons of the failure of previous work [7, 8] to demonstrate genetic causal link to metastasis. The first one is the molecular heterogeneity of cancer specimen studied. Metastatic lesions (MT) have been shown to harbour from less than 10 to more than 800 somatic mutations in the exomic region [7]. The molecular features of primary tumours are also highly inconsistent which led to selection of distinct subclasses [9]. There may be multiple paths leading to dissemination into distant locations for each subclass of primary tumour, making published studies underpowered. Secondly, metastasis may be purely stochastic process, independent of specific genetic traits present in the primary lesion. Factors outside cancerous cells, like immunological response, relative position of primary tumour in respect to existing vasculature and susceptibility of vascular epithelia to invasion may contribute to metastasis, greater than any single genetic mutation.

There are three aspects of metastasis genetics that are yet to be explained: which alterations are key drivers of the process, in what mechanism they occur and what functions/aspects of cell do they modify. The first problem is complicated by the fact that multiple distinct DNA modifications can lead to similar phenotype, which increases sample size required to prove causal link. Functional alterations are yet impossible to decrypt on genomic scale with genotype alone and without broad information on gene expression.

Here we employ next generation sequencing for both, exome genotyping and transcriptome sequencing of freshly frozen samples sets (normal tissue, primary tumour and liver metastasis) from 7 patients to characterise mutational landscape of metastatic CRC.

## Methods

### Tissue specimen

Primary colon tumours with normal tissue margin and slices of liver metastases less than 1 mm thick and less than 10 mm long were dissected simultaneously. Parts of both were used for immediate pathology examination and the rest was frozen in -80 °C upon further processing.

For primary tumours, sections of uniformly malignant tissue were selected in macro-dissection procedure. For 5 primary tumours further dissection of multiple spatially separated fragments of malignant tissue was conducted to assess intra-tumour variability. For three primary tumours microdissection was performed using PALM laser microdissection and pressure catapulting (LMPC) system (PALM MicroBeam with PALM RoboMover module and PALM RoboSoftware; Carl Zeiss MicroImaging GmbH, Germany) (samples 10PT3, 10PT4, 5PT1, 5PT2, 9PT4, 9PT5).

The extraction and purification of DNA was performed using QIAamp DNA Micro Kit (Qiagen, Germany) according to Protocol for Isolation of Genomic DNA from Laser-Microdissected Tissues. DNA sample concentration was measured using NanoDrop spectrophotometer, following the manufacturer’s instructions. DNA was further stored at -20 °C.

### Exome sequencing

Exome libraries were generated using Nextera Rapid Capture Expanded Exome Enrichment Kit (Illumina). Sequencing (2x94bp or longer) was performed using Illumina HiSeq 2500 system with TruSeq PE Cluster Kit v3 and SBS Kit v3(Illumina). The sequencing quality was evaluated with FastQC (http://www.bioinformatics.babraham.ac.uk/ projects/fastqc). Sequences were obtained using the Solexa Analysis Pipeline and mapped to the human genome assembly (hg19) using Bowtie2 (version 4.1.2 [10]). Variants differentiating tumours and respective normal tissue were called using Varscan2 (version 2.3.7 [11]). Short and medium structural variants were detected using Pindel (version 0.2.4t [12]). Called single nucleotide variants were filtered with fpfilter (https://github.com/ckandoth/variant-filter/blob/master/fpfilter.pl) using default parameters with the exception of minimal allele fraction set to 0.1 (min-var-frac = 0.1).

Annovar (version 20,150,617 [13]) was used to annotate variants with genes, position respective to genes (exonic/intronic/splicing/untranslated region (UTR)/ upstream/ downstream/ intergenic), impact on protein sequence (synonymous/ nonsynonymous/ stopgain/ stoploss) and identify variants previously linked to CRC development according to International Cancer Genome Consortium (ICGC, version 21). Frequencies of minor allele in the 1000 Genomes Project database, Exome Sequencing Project of National Heart, Lung, and Blood Institute (6500 exomes, [14]) and in Exome Aggregation Consortium database (ExAC, > 60,000 exomes, [15]) were also annotated using Annovar. To exclude common variants, homozygous non-reference variants present in more than 50% population according to ExAC database were removed.

Variants previously linked to CRC development were imported from COSMIC database (version 20,161,128 [5]).

“Filtered variants” sets were created in three consecutive steps. First, variants differentiating tumour and normal tissue were called with Varscan2. Detected variants were then filtered according to read depth (> = 20) and number of non-reference reads from each strand (> = 4). Last, variants detected in more than 1% of population according to ExAC, 6500 exomes or 1000 Genomes Project (both global and European) database were discarded. Exclusive metastatic variants (EMV) were selected in similar way, by further removing variants detected in primary tumours from metastatic variants set.

Functional analysis of EMV was performed with Gene Set Enrichment Analysis (GSEA) software (version 2.2.4, [16]), using Reactome [17] as gene sets database. Two scores were used as gene rankings for GSEA – Cancer-specific High-throughput Annotation of Somatic Mutations (CHASM) [18] for missense driver cancer mutations and highest Combined Annotation Dependent Depletion (CADD) score variant per gene [19] for all the variants.

### Transciptome sequencing

Total RNA was isolated from tissue using RNeasy Plus Mini Kit (Qiagen, Germany), following manufacturer protocol. The purity and quantity of RNA was measured with NanoDrop spectrophotometer and assessed using an Agilent 2100 Bioanalyzer with RNA 6000 Nano Kit (Agilent, California). Samples were stored at -70 °C.

Sequencing libraries were generated using Ion AmpliSeq Library Kit Plus (Thermo Fisher). Sequencing was performed using Ion Proton instrument with 5 or 6 samples per chip with Ion PI Hi-Q Sequencing 200 Kit (Thermo Fisher). Reads were aligned to the hg19 AmpliSeq Transcriptome ERCC v1, target panel 21 K v1. Transcripts were quantified with HTseq-count (version 0.6.0 [20]), run with default options. Differentially expressed genes were determined with negative binominal test implemented in DESeq2 package (version 1.12.4, [21]). Patients were used as confounding variable. *P*-values were corrected for multiple hypotheses testing with Benjamini-Hochberg procedure and differences with corrected *p*-values < 0.05 were considered significant.

Overrepresentation of Gene Ontology (GO) terms [22] assigned to genes with the most marked expression differences between groups was tested with Fisher Exact test implemented in the GOstats package (version 2.40.0, [23]). Tests were performed in the “conditional” mode, separately for biological process and molecular function branch. Only terms with more than 2 and less than 2% of the total number of observed transcribed genes (~ 20,000) were assessed. P-values from Fisher Exact test were corrected with Benjamini-Hochberg procedure.

The link between transcriptome changes and observed mutations was probed with Kolmogorov-Smirnov test. Genes were sorted according to expression fold-change (FC) between each tumour sample and respective normal sample. Positions of genes carrying selected classes of mutations on FC sorted list was used as an input for Kolmogorov-Smirnov test. Analysis was done separately for all non-silent, homozygous non-silent, stopgain and indel mutations.

### Availability of data and materials

The dataset supporting the conclusions of this article is available in the Gene Expression Omnibus repository (https://www.ncbi.nlm.nih.gov/geo/) under entry GSE89393.

## Results

### Exome sequencing

**Fig. 1:**
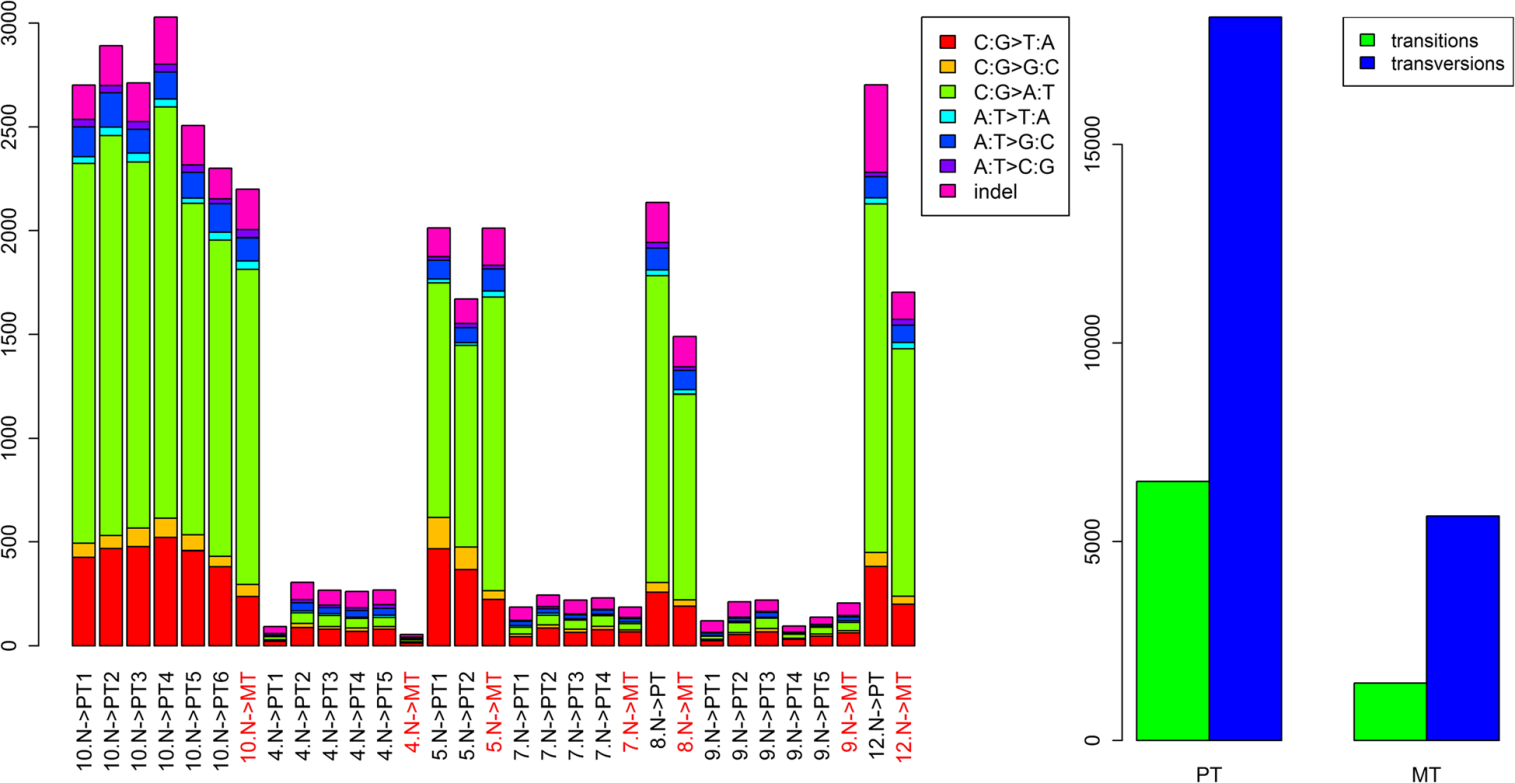
Mutation types in freshly frozen samples. N- > PT/MT - mutations differentiating primary/metastatic tumour (PT/MT) and respective norm. Transitions and transversions are given total for all PT/MT samples

Between 1.5 and 9.7 billion base reads that mapped to the reference genome were generated during exome sequencing for 7 sets of freshly frozen samples (Additional file 1: Table S1). Each set consisted of normal tissue, metastatic tumour and between 1 and 6 samples of primary tumour. Between 54 and 3029 variants differentiating primary tumours and metastases from normal tissue were found (“filtered variants”, Fig. 1). Between 1 and 88 of those variants were stop-gains. Samples could be classified into low and high mutation count categories with between 54 and 306 mutations detected in the former and between 1490 and 3029 in the latter (1–7 and 36–88 stop-gains, respectively). Characteristics of variants detected in metastatic samples closely resembled those of respective primary tumours. 426 filtered variants were detected in more than one patient, 49 were detected in 3 patients and 17 in 4 patients (Additional file 2: Table S2). There were three frameshift substitutions detected simultaneously in 4 patients, in ABTB2, TPI1 and GLI2. 6 filtered variants that were homozygous, exonic and nonsynonymous were detected in at least two patients (Additional file 3: Table S3). Frameshift causing insertion of adenine at codon 336 of transcript NM_000365 of TPI1 was detected in four patients, three of those insertions were homozygous.

Mutations of C:G pairs were detected over nine times more often than mutations of A:T pairs and three times as many transversions than transitions (Fig. 1). Most of the filtered variants were exonic (46.2%), intronic (14.5%), 3’UTR (11.6%) or intergenic (10.5%), which was in line with library preparation method used. 29.0% of filtered variants were nonsynonymous SNV, 11.4% were synonymous SNV and 2.6% were stopgains according to Annovar (Additional file 4: Table S4).

Numerous variants in genes already implicated in CRC development were detected among filtered variants (Additional file 5: Table S5). Mutation in KRAS was detected in five patients and mutation in APC, POLE and PTPRT was detected in four patients. Notably there were no mutations detected in APC and KRAS in 3 metastatic samples although primary tumours from the same patients were carrying mutations in this genes.

In metastases there were between 26 and 2029 variants that weren’t detected in any normal tissue nor in primary tumours (exclusive metastatic variants - EMV). Mutation types were similar to those differentiating primary tumours and normal tissue with C:G pairs substitutions ten times more likely than A:T pair and 4.6 times as many transversions than transitions (Additional file 6: Fig. S2). 47.3% of EMV were exonic, 15.7% intronic, 10.6% 3’UTR and 10.3% were intergenic. 30.9% EMV were nonsynonymous SNV, 10.9% were synonymous SNV and 2.8% were stopgain (Additional file 7: Table S6). The most frequently mutated genes in MT (normalized for length) were NHLH2, RPL13A and SSNA1.

Among variants exclusive to metastatic tumors (EMVs), 89 missense variants are potential cancer drivers (FDR(false discovery rate)-adjusted CHASM *p*-value < 0.05). Only one variant, BAHD1 p. R533S is present in more than one sample (Additional file 8: Table S7). There are 128 genes, are potential cancer-driver genes (FDR-adjusted CHASM composite p-value < 0.05). None of them is mutated in every sample – the most changed is *PLXND1,* with mutation in 5 samples (Additional file 9: Table S8). CELSR3 had EMV in four patients, BAHD1 and PNPLA6 in three. GSEA analysis with CHASM score as ranking feature revealed 5 Reactome pathways with FDR values in 0.05–0.1 range, which included Signaling by FGFR pathway (Additional file 10: Table S9). On the other hand, similar analysis with highest CADD score per gene yielded no significant results (not shown).

### Transcriptome sequencing

Between 7.8 and 22.4 million of tags read during transcriptome sequencing were mapped to the reference sequence. Between 61.2 and 74.5% of the reference transcripts were detected (Additional file 11: Table S10). There were two outliers among samples according to Principal Component Analysis (PCA), (Additional file 12: Fig. S1). Corresponding samples weren’t taken into account in comparisons between groups.

Expression of 3066 genes was significantly different between normal tissue and primary tumours. 1555/1511 were up/down regulated in tumours. 2677 of them showed at least 2 fold change and 216 over 10 fold change in expression (Additional file 13: Table S11A). There were genes with over 100 fold decrease and over 100 fold increase in expression (Table 1). The most notable examples of down-regulated genes were GUCA2B (FC = 187), TMIGD1 (FC = 129) and CA1 (FC = 121), while CST1 and S100A2 (FC = 131/88.7, respectively) were highly expressed in tumours but not in normal tissue. Differences in expression were attributed to electrolyte homeostasis (GO:0015711, GO:0006811) and some metabolic processes, including lipid and fatty acid metabolism (Table 3A). “Response to drug” (GO:0042493) is particularly interesting in this context because all samples were collected prior to chemotherapeutic treatment. “Magnesium ion binding” (GO:0000287) was the only molecular function overrepresented among the most differentiating genes (adj. *p* = 0.015, FC = 1.98).

**Table 1:** Genes with the most significant differences in expression between normal colon and primary tumours

RefSeq ID	gene symbol	gene name	FC	adjusted *p*-value
NM_001898	CST1	cystatin SN	131.6	3E-25
NM_005978	S100A2	S100 calcium binding protein A2	88.7	1.45E-27
NM_182507	KRT80	keratin 80	52.9	8.81E-21
NM_178493	NOTUM	NOTUM, palmitoleoyl-protein carboxylesterase	46.6	3.01E-17
NM_005069	SIM2	single-minded family bHLH transcription factor 2	44.2	1.15E-18
NM_001201	BMP3	bone morphogenetic protein 3	0.0217	3.34E-15
NM_182546	VSTM2A	V-set and transmembrane domain containing 2A	0.0170	6.77E-14
NM_001169	AQP8	aquaporin 8	0.0169	3.01E-17
NM_001285	CLCA1	chloride channel accessory 1	0.0164	5.46E-17
NM_152338	ZG16	zymogen granule protein 16	0.0142	2.53E-14
NM_000067	CA2	carbonic anhydrase 2	0.0135	2.33E-42
NM_005182	CA7	carbonic anhydrase 7	0.0129	9.63E-16
NM_001134742	SLC4A4	solute carrier family 4 member 4	0.0120	2.19E-27
NM_001128831	CA1	carbonic anhydrase 1	0.0083	9.96E-18
NM_206832	TMIGD1	transmembrane and immunoglobulin domain containing 1	0.0078	7.55E-21
NM_007102	GUCA2B	guanylate cyclase activator 2B	0.0053	1.03E-30

FC - fold change (expression in primary tumours/expression in normal colon)

105 genes were differentially expressed between metastases and primary tumours. 38/67 were up/down regulated in metastases. For CRP and FGG expression increased over 50-fold (Table 2, Additional file 13: Table S11B). The most overrepresented biological processes among differentiating genes were cellular component and extracellular matrix organization, followed by immune response-related processes (Table 3B). Interestingly, neither EGFR nor EGF, previously proposed as essential for matrix organization [24], were found to be differentially expressed. The most significant molecular function was “heparin binding” and several extracellular matrix remodelling processes (Additional file 14: Table S12).

**Table 2:** Genes with the most significant differences in expression between primary tumours and metastases

RefSeq ID	gene symbol	gene name	FC	adjusted p-value
NM_000567	CRP	C-reactive protein	85.0	2.36E-21
NM_021870	FGG	fibrinogen gamma chain	56.0	1.98E-12
NM_001204307	GC	GC, vitamin D binding protein	29.1	1.19E-08
NM_002216	ITIH2	inter-alpha-trypsin inhibitor heavy chain 2	26.1	1.43E-06
NM_005141	FGB	fibrinogen beta chain	25.1	4.18E-05
NM_000042	APOH	apolipoprotein H	22.2	4.18E-05
NM_002215	ITIH1	inter-alpha-trypsin inhibitor heavy chain 1	22.0	1.81E-05
NM_000607	ORM1	orosomucoid 1	16.0	8E-06
NM_004467	FGL1	fibrinogen like 1	14.2	0.003387
NM_001063	TF	transferrin	12.9	0.000902
NM_000505	F12	coagulation factor XII	12.3	0.003333
NM_145285	NKX2–3	NK2 homeobox 3	0.055	2.63E-07
NM_003480	MFAP5	microfibrillar associated protein 5	0.068	6.59E-05
NM_004950	EPYC	epiphycan	0.070	0.001189
NM_002148	HOXD10	homeobox D10	0.071	7.38E-05
NM_001170807	FHL5	four and a half LIM domains 5	0.078	4.18E-05
NM_001145311	PLIN1	perilipin 1	0.080	0.001189
NM_019849	SLC7A10	solute carrier family 7 member 10	0.080	0.00042

FC - fold change (expression in metastases/expression in primary tumours)

**Table 3:** GO terms significant for normal colon - primary tumour - metastasis transition

GO ID	GO name	adjusted p-value	count	expected count
A				
GO:0015711	organic anion transport	3.64E-03	51	25.2
GO:0006811	ion transport	3.64E-03	44	20.8
GO:0034440	lipid oxidation	6.15E-03	27	10.5
GO:0061326	renal tubule development	2.46E-02	24	9.7
GO:0006730	one-carbon metabolic process	2.46E-02	11	2.7
GO:0042493	response to drug	2.46E-02	54	30.6
GO:0006635	fatty acid beta-oxidation	4.01E-02	12	3.3
GO:0030214	hyaluronan catabolic process	4.13E-02	8	1.6
GO:0072163	mesonephric epithelium development	4.13E-02	24	10.3
B				
GO:0016043	cellular component organization	1.03E-03	42	18.3
GO:0030198	extracellular matrix organization	3.29E-03	40	18.1
GO:0002576	platelet degranulation	3.72E-03	28	10.9
GO:0006953	acute-phase response	3.72E-03	17	4.9
GO:0034367	macromolecular complex remodeling	1.67E-02	11	2.6
GO:0010951	negative regulation of endopeptidase activity	1.81E-02	27	11.5
GO:0044057	regulation of system process	1.81E-02	60	35.1
GO:0007204	positive regulation of cytosolic calcium ion concentration	1.81E-02	45	24.0
GO:0006559	L-phenylalanine catabolic process	2.45E-02	7	1.2

GO biological processes with the highest overrepresentation in the 10% of genes with the lowest *p*-value (selected subset) in comparison between normal colon vs primary tumour (A) or primary tumour vs metastases (B). Count - number of genes in selected subset attributed to a given GO term. Expected count - number of genes expected to be attributed to given category by chance.

The aggregated effect of accumulated mutations was visible in the observed transcriptome remodelling. When genes were sorted according to fold-change of expression (FC) for three pairs of tumour-normal sample, the genes with detected filtered variants weren’t distributed randomly. For various classes of filtered variants there was a significant bias of distribution along FC-sorted genes detected with Kolmogorov-Sminov test (bold highlight in Table 4). Differences in one MT transcriptome vs respective normal tissue were linked to the set of all non silent mutations. Interestingly, stop-gains were less impactful on their own, with significant association with transcriptome changes only in one primary and none of metastatic tumours (Table 4).

**Table 4:** Assessment of transcription changes of genes with given alterations in the coding sequences

Sample ID	10.PT1	10.PT2	10.PT3	10.PT4	10.PT5	10.PT6	10.MT	5.MT	7.PT1	7.PT2	7.PT3	7.PT4	7.MT	12.PT	12.MT
stopgain	0.6	0.9	0.6	**1.9**	0.3	**1.4**	0.2	0.3	0.1		0.1			1.1	0.4
non silent	**7.4**	**8.1**	**7.5**	**8.8**	**4.5**	**6.9**	0.7	0.5	0.5	0.0	0.2	1.3	0.4	**Inf**	**2.1**
silent exonic	**1.7**	1.2	**3.8**	**4.4**	1.1	**3.0**	0.1	0.3	1.1	0.3	0.4	0.6	**1.5**	**8.2**	1.2
indels all	0.2	0.6	0.8	0.2	1.0	0.7	0.3	0.3	**3.3**	0.5	0.4	0.3	0.0	**2.3**	0.1

Given values are -log10 of p-value from Kolmogorow-Smirnoff test of altered vs non-altered genes (see methods). Bold highlights significant association (values greater than -log10(0.05)). Inf - values greater than 10

## Discussion

Contrary to previously published results [7], where transversions were less prevalent than transitions by twofold, there were 3 times more transversions than transitions. The number of detected somatic variants was, on average, more than two times higher here than in Lim B et al. [7]. Discrepancies cannot be explained neither by sequencing technology (Illumina HiSeq in both cases), nor by sequencing depth, which was similar (101 vs 133). Mapping software was also comparable (BWA [25] vs Bowtie 2). The most significant protocol difference is that we used Varscan2 [11] instead of MuTect [26]. Varscan2 is more sensitive than MuTect, detecting over 3 times more SNP in some scenarios [27]. Furthermore, MuTect misses some high quality variants [28]. We believe that Mutect is overly conservative, especially when sequencing depth is high (> 100). Additional filtering for minimal number of reads from each strand (> = 4) supporting variant protect against high false-positive rate. Contradicting results on mutation type distribution highlight the dependence of conclusions regarding mutation mechanism on analytic choices.

There were 89 cancer-driver mutations among EMVs predicted by CHASM, however most of them concerned only one tumour. On the other hand, on gene level there were 128 cancer-driver genes predicted, two mutated in four patients and one in five. Moreover, GSEA analysis revealed significant enrichment of FGFR signalling and antigen processing pathways. These results suggest indeed there are no specific mutations involved in metastatic processes, however the cancer-driver mutation distribution is not entirely random since it involves specific genes and pathways.

High levels of CRP, the gene with the most significant expression increase in liver metastases (Table 2), were previously associated with poorer prognosis for CRC [29, 30]. This is in line with other findings associating various inflammation symptoms with metastasis (Table 3) [31]. The key players in inflammation progression are matrix metalloproteinases (MMP) well described in the CRC context [32] and significantly differentiating primary tumour from normal tissue here (Additional file 13: Table S11). EGFR was labelled MMP regulator [24] and was found downregulated in lymph node metastasis vs primary tumours [33]. In our study neither EGF nor EGFR expression did differentiate metastasis from primary tumour, which suggests there is other mode of MMP activation.

## Conclusions

Seven sample sets are, like in previous work [7, 8], not enough to prove any direct genetic linkage to metastatic process. Transcriptome sequencing however, revealed some tissue remodelling and immune processes essential for metastasis (Table 3, Additional file 14: Table S12). Furthermore, we were able to associate observed remodelling of transcription in both primary and metastatic tumours with accumulated mutations (Table 4). This supports the thesis that widespread genetic instability generates the environment for evolutional selection of tumour cells and is the driver of malignancy.

## Additional files

Additional file 1: **Table S1.** Sequencing parameters for 3 samples with the highest and 3 samples with the lowest sequencing yield, along with the mean and median of sequencing parameters for all (31) samples. (DOCX 12 kb)

Additional file 2: **Table S2.** Filtered variants detected in three or more patients. Chromosome, start, end - genome coordinates of variant; Ref/Alt - reference/alternative variant sequence. GT - genotype detected in a given sample (0 - reference, 1-alternative). DP - number of high quality reads at a given position in a given patient. Func.refGene - location of variant relative to gene. Gene - symbol of a gene the given variant maps to, or names of genes the given variants maps in between. GeneDetail.refGene - refSeq gene ID or distance to nearest gene given in the “Gene” column. ExAC_XXX - frequency of the alternative variant in XXX population according to ExAC database. 1000g_all/eur - variant frequency in the 1000 Genomes Project database (total/European). esp6500siv2_all - variant frequency according to National Heart, Lung, and Blood Institute GO Exome Sequencing Project. SIFT/Polyphen2/LRT/FATHMM/RadialSVM “_pred” - prediction of variant impact on protein structure: B-benign, N-neutral, T-tolerated, D-deleterious. ICGC_Id - variant ID in ICGC database (known cancer-related variants). Heterozygous variants are marked orange, homozygous are marked red. (XLSX 34 kb)

Additional file 3: **Table S3.** Homozygous, exonic and nonsynonymous filtered variants that were detected in more than one patient. Column description is the same as in Additional file 2: Table S2. (XLSX 11 kb)

Additional file 4: **Table S4.** Functional impact of filtered variants on protein coding sequences. (XLSX 9 kb)

Additional file 5: **Table S5.** Filtered variants detected in genes implicated in CRC development according to COSMIC database. Four consecutive panels describe zygosity (“GT.sample_name”), impact on protein structure and function (“ExonicFunc.sample_name”), sequencing depth (“DP.sample_name”) and genomic position (“POS.sample_name”). Each field may contain more than one entry if multiple variants were detected in one gene. Red/orange fields denotes homozygous/heterozygous variants. If one of multiple variants was homozygous given field was marked red. Primary/metastatic samples were marked yellow/red, respectively. (XLSX 27 kb)

Additional file 6: **Figure S2.** Mutation types in EMV in freshly frozen samples. Transitions and transversions are given total for all single nucleotide substitutions. (TIFF 427 kb)

Additional file 7: **Table S6.** Functional impact of EMV on protein coding sequences. (XLSX 9 kb)

Additional file 8: **Table S7.** EMV Cancer driver mutations according to CHASM algorithm. Chrom – chromosome number, Position – mutation position, Ref – reference base, Alt – variant base, Sample.ID – sample with mutation, HUGO.symbol – HUGO gene symbol, Protein.sequence.change – amino-acid change. CHASM.p.value – *p*-value for CHASM, dbSNP – identifier in dbSNP, 1000.Genomes/ ESP6500/ ExAC – allele frequencies in different exome-sequencing projects, COSMIC.ID – COSMIC identifier, Occurrences.in.COSMIC.by.primary.sites – organs, harboring somatic mutations in this gene in COSMIC database, ClinVar.Clinical.Significance – ClinVar clinical significance, Number.of.samples – number of samples with mutation, Qvalue – CHASM.p.value corrected for multiple testing with FDR method (XLSX 291 kb)

Additional file 9: **Table S8.** CHASM cancer-driver identification results for whole genes, where HUGO.symbol – HUGO gene symbol, Number.of.variants – number of variants per gene, Most.severe.sequence.ontology – most severe mutation consequence within gene, CHASM.score – CHASM score for whole gene, CHASM.composite.p.value – composite *p*-value for whole gene, Qvalue - CHASM.composite.p.value corrected for multiple testing with FDR method, Driver.genes – whether a gene is a driver or tumor-suppressor gene (TSG), Occurrences.in.COSMIC.by.primary.sites – organs, harboring somatic mutations in this gene in COSMIC database, Number.of.samples – number of samples with driver mutation in this gene, TCGA.Mutation.Cluster – whether a TCGA mutation cluster is present within a gene. (XLSX 186 kb)

Additional file 10: **Table S9.** GSEA results in which CHASM score for a gene was taken as a ranking metric where NAME – Reactome pathway name, SIZE – size of a dataset after substraction of genes not present in ranked set, ES/NES – enrichment score/normalized enrichment score, NOM p-value – nominal p-value, FDR – false discovery rate, LEADING EDGE – statistics used to define the leading edge subset (for details, please refer to http://software.broadinstitute.org/gsea/doc/GSEAUserGuideTEXT.htm#_GSEA_Report). (XLSX 15 kb)

Additional file 11: **Table S10.** Sequencing summary for the transcriptome profiling. Valid read - fraction of reads meeting quality standard, on target - fraction of valid reads mapping to the part of genome targeted by employed primers. (XLSX 9 kb)

Additional file 12: **Figure S1.** Plot of the first four principal components (PC) for gene expression according to RNA-Seq. Result for all samples (A, top panels) and after removal of two outliers (B, bottom panels). All axes depict arbitrary units. (TIF 1016 kb)

Additional file 13: **Table S11.** Results of RNA expression comparison between normal colon tissue and primary tumours (A) and between primary tumours and metastases (B). Target - the producers (Life Technologies) id of respective amplicon; padj - p-value adjusted for multiple hypotheses testing with Benjamini-Hochberg algorithm. (XLSX 2856 kb)

Additional file 14: **Table S12.** GO terms (molecular function branch) with the highest overrepresentation in the 10% of genes with the lowest p-value (“top genes”) in the comparison between normal vs PT (A) and PT vs MT (B). Count - number of genes associated to the given GO term in the “top genes” set according to p-value in a given comparison, expected count - number of genes expected to be associated to the given GO term by chance in the “top genes” set. (XLS 25 kb)

## Abbreviations

CADD: Combined Annotation Dependent Depletion
CHASM: Cancer-specific High-throughput Annotation of Somatic Mutations
COSMIC: Catalogue Of Somatic Mutations In Cancer
CRC: colorectal cancer
DP: sequencing depth
EMV: exclusive metastatic variants
ExAC: Exome Aggregation Consortium database
FC: fold-change
FDR: false discovery rate
GO: Gene Ontology
GSEA: gene set enrichment analysis
GT: genotype
ICGC: International Cancer Genome Consortium
LMPC: laser microdissection and pressure catapulting
MMP: matrix metalloproteinases
MT: metastatic tumour
PCA: principal component analysis
PT: primary tumour
UTR: untranslated region

## References

[1] Elferink MAG, de Jong KP, Klaase JM, Siemerink EJ, de Wilt JHW (2015). Metachronous metastases from colorectal cancer: a population-based study in north-East Netherlands. Int J Color Dis.

[2] Gupta GP, Massagué J (2006). Cancer metastasis: building a framework. Cell.

[3] Langley RR, Fidler IJ (2011). The seed and soil hypothesis revisited--the role of tumor-stroma interactions in metastasis to different organs. Int J Cancer.

[4] Grothey A (2008). Bevacizumab beyond first progression is associated with prolonged overall survival in metastatic colorectal cancer: results from a large observational cohort study (BRiTE). J Clin Oncol Off J Am Soc Clin Oncol.

[5] Forbes SA (2015). COSMIC: exploring the world’s knowledge of somatic mutations in human cancer. Nucleic Acids Res.

[6] Vanharanta S, Massagué J (2013). Origins of metastatic traits. Cancer Cell.

[7] Lim B (2015). Genome-wide mutation profiles of colorectal tumors and associated liver metastases at the exome and transcriptome levels. Oncotarget.

[8] Zehir A (2017). Mutational landscape of metastatic cancer revealed from prospective clinical sequencing of 10,000 patients. Nat Med.

[9] Guinney J (2015). The consensus molecular subtypes of colorectal cancer. Nat Med.

[10] Langmead B, Salzberg SL (2012). Fast gapped-read alignment with bowtie 2. Nat Methods.

[11] Koboldt DC (2012). VarScan 2: somatic mutation and copy number alteration discovery in cancer by exome sequencing. Genome Res.

[12] Ye K, Schulz MH, Long Q, Apweiler R, Ning Z (2009). Pindel: a pattern growth approach to detect break points of large deletions and medium sized insertions from paired-end short reads. Bioinforma. Oxf. Engl..

[13] Wang K, Li M, Hakonarson H (2010). ANNOVAR: functional annotation of genetic variants from high-throughput sequencing data. Nucleic Acids Res.

[14] Sudmant PH (2015). An integrated map of structural variation in 2,504 human genomes. Nature.

[15] Lek M (2016). Analysis of protein-coding genetic variation in 60,706 humans. Nature.

[16] Subramanian A (2005). Gene set enrichment analysis: a knowledge-based approach for interpreting genome-wide expression profiles. Proc Natl Acad Sci U S A.

[17] Fabregat A (2016). The Reactome pathway knowledgebase. Nucleic Acids Res.

[18] Carter H (2009). Cancer-specific high-throughput annotation of somatic mutations: computational prediction of driver missense mutations. Cancer Res.

[19] Kircher M, Witten DM, Jain P, O’Roak BJ, Cooper GM, Shendure J (2014). A general framework for estimating the relative pathogenicity of human genetic variants. Nat Genet.

[20] Anders S, Pyl PT, Huber W (2015). HTSeq--a Python framework to work with high-throughput sequencing data. Bioinforma. Oxf. Engl..

[21] Love MI, Huber W, Anders S (2014). Moderated estimation of fold change and dispersion for RNA-seq data with DESeq2. Genome Biol.

[22] Ashburner M (2000). Gene ontology: tool for the unification of biology. The gene ontology consortium. Nat Genet.

[23] Falcon S, Gentleman R (2007). Using GOstats to test gene lists for GO term association. Bioinforma Oxf Engl.

[24] Kajanne R (2007). EGF-R regulates MMP function in fibroblasts through MAPK and AP-1 pathways. J Cell Physiol.

[25] Li H, Durbin R (2010). Fast and accurate long-read alignment with burrows-wheeler transform. Bioinforma. Oxf. Engl..

[26] Cibulskis K (2013). Sensitive detection of somatic point mutations in impure and heterogeneous cancer samples. Nat Biotechnol.

[27] Krøigård AB, Thomassen M, Lænkholm A-V, Kruse TA, Larsen MJ (2016). Evaluation of nine somatic variant callers for detection of somatic mutations in exome and targeted deep sequencing data. PLoS One.

[28] Wang Q (2013). Detecting somatic point mutations in cancer genome sequencing data: a comparison of mutation callers. Genome Med.

[29] Shrotriya S, Walsh D, Bennani-Baiti N, Thomas S, Lorton C (2015). C-reactive protein is an important biomarker for prognosis tumor recurrence and treatment response in adult solid tumors: a systematic review. PLoS One.

[30] Li C, Xu Q, Chen L, Luo C, Ying J, Liu J (2017). C-reactive protein (CRP) as a prognostic factor for colorectal cancer after surgical resection of pulmonary metastases. Bull Cancer (Paris).

[31] Terzić J, Grivennikov S, Karin E, Karin M (2010). Inflammation and colon cancer. Gastroenterology.

[32] Said AH, Raufman J-P, Xie G (2014). The role of matrix metalloproteinases in colorectal cancer. Cancers.

[33] Xie N, Yao Y, Wan L, Zhu T, Liu L, Yuan J (2017). Next-generation sequencing reveals lymph node metastasis associated genetic markers in colorectal cancer. Exp Ther Med.

