# Supplementary material for: Exome scale map of genetic alterations promoting metastasis in colorectal cancer
Source: BMC Genet. 2018 Sep 19;19:85. doi: 10.1186/s12863-018-0673-0 (PMC6146521; doi:10.1186/s12863-018-0673-0)
Supplement: Supplementary file 1 — Table S1. Sequencing parameters for 3 samples with the highest and 3 samples with the lowest sequencing yield, along with the mean and median of sequencing parameters for all (31) samples. (DOCX 12 kb) [file 12863_2018_673_MOESM1_ESM.docx]

| sample | 5PT1 | 5PT2 | 12PT | 4PT1 | 4MT | 9N | **mean**  **(all samples)** | **median**  **(all samples)** |
| --- | --- | --- | --- | --- | --- | --- | --- | --- |
| Total base reads (10^9) | 17.5 | 18.4 | 16.3 | 3.7 | 3.1 | 3.4 | 9.9 | 10.5 |
| Total base reads on target (10^9) | 9.3 | 9.7 | 9.2 | 1.8 | 1.5 | 1.7 | 5.2 | 6.0 |
| Mean coverage | 205 | 214 | 203 | 40 | 34 | 36 | 115 | 133 |
| Target base coverage at 1x | 0.71 | 0.73 | 0.65 | 0.81 | 0.80 | 0.80 | 0.71 | 0.73 |
| Target base coverage at 20x | 0.63 | 0.65 | 0.56 | 0.57 | 0.52 | 0.54 | 0.57 | 0.56 |
| Target base coverage at 100x | 0.44 | 0.45 | 0.39 | 0.10 | 0.07 | 0.08 | 0.29 | 0.33 |
| Target base coverage at 500x | 0.13 | 0.14 | 0.13 | 0.00 | 0.00 | 0.00 | 0.05 | 0.04 |

Supplementary table 1. Sequencing parameters for 3 samples with the highest and 3 samples with the lowest sequencing yield, along with the mean and median of sequencing parameters for all freshly-frozen samples.
