# Supplementary figures and images for: Exome scale map of genetic alterations promoting metastasis in colorectal cancer
Source: BMC Genet. 2018 Sep 19;19:85. doi: 10.1186/s12863-018-0673-0 (PMC6146521; doi:10.1186/s12863-018-0673-0)

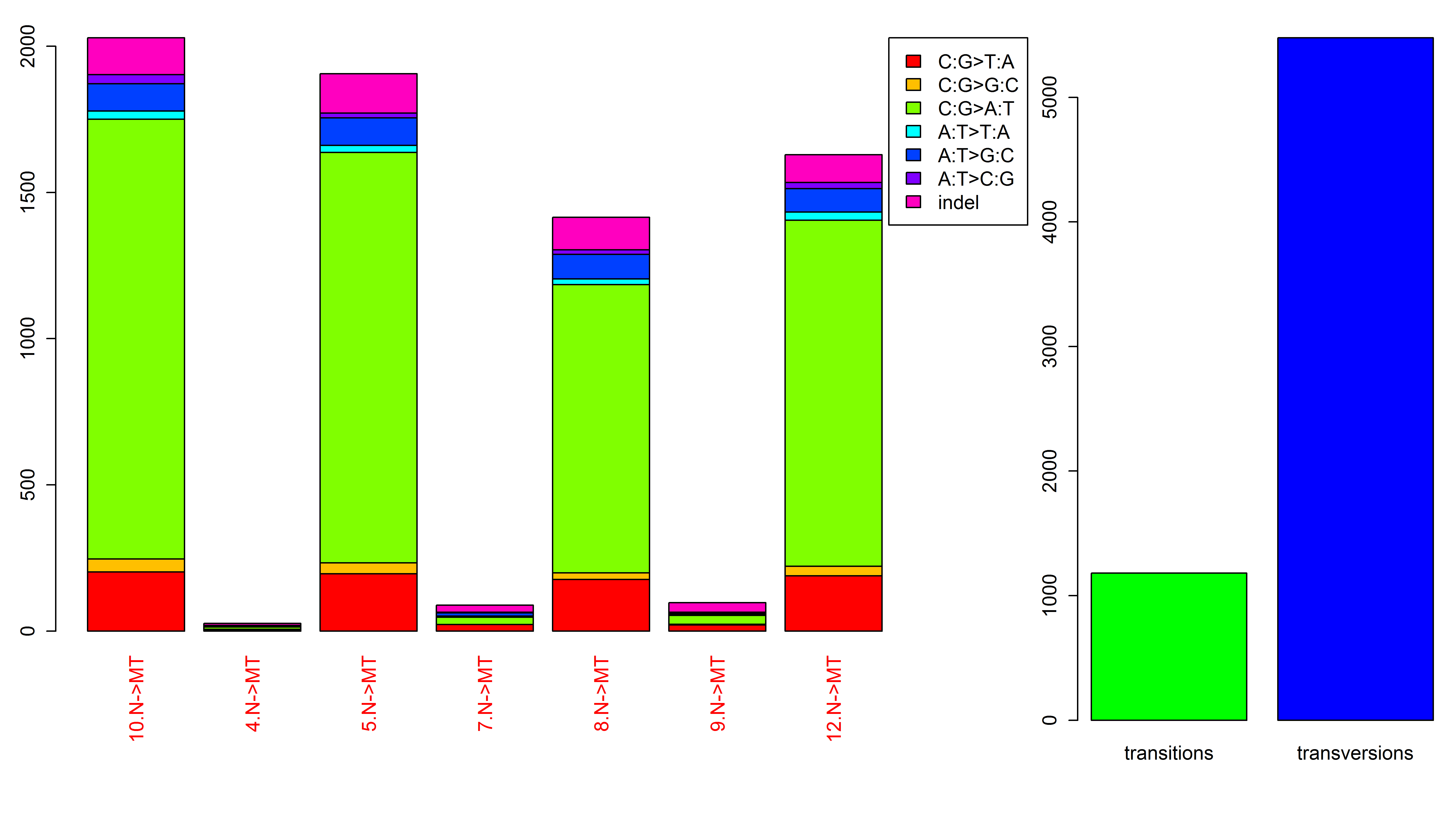

Supplement: Supplementary file 6 — Figure S2. Mutation types in EMV in freshly frozen samples. Transitions and transversions are given total for all single nucleotide substitutions. (TIFF 427 kb) [file 12863_2018_673_MOESM6_ESM.tiff]
